# Transanal Irrigation for Refractory Chronic Idiopathic Constipation: Patients Perceive a Safe and Effective Therapy

**DOI:** 10.1155/2017/3826087

**Published:** 2017-01-01

**Authors:** Kevin J. Etherson, Ian Minty, Iain M. Bain, Jeremy Cundall, Yan Yiannakou

**Affiliations:** ^1^Department of Colorectal Surgery, County Durham and Darlington NHS Foundation Trust, Durham, UK; ^2^Department of Radiology, County Durham and Darlington NHS Foundation Trust, Durham, UK

## Abstract

*Background.* Transanal irrigation (TAI) can successfully treat neurogenic bowel dysfunction (NBD), but patient perception of its use in chronic idiopathic constipation (CIC) is unknown.* Objective.* To evaluate patient perceptions of the efficacy and safety of TAI for CIC and whether there are predictive factors of perceived treatment response.* Methods.* Prospective data collection of baseline physiology and symptom severity; retrospective evaluation of efficacy and safety perceptions using a snapshot survey. All patients fulfilling the Rome III criteria for functional constipation with chronic idiopathic aetiology were included. The main outcome measure was the duration of patients' usage of TAI.* Results.* 102 patients reported 21,476 irrigations over 119 patient years, with a mean duration of therapy use of 60.5 weeks [SD 73.2 : SE 7.3]. Overall symptom improvement included general well-being (65%), rectal clearance (63%), bloating (49%), abdominal pain (48%), and bowel frequency (42%). 68 patients (67%) were “moderately better” or “very much better” on a satisfaction question. Reported complications were minor. No correlation was demonstrated between duration of therapy use and baseline measures.* Conclusion.* A significant proportion of CIC sufferers use TAI as a long-term or bridging therapy and perceive it as safe. This therapy demands a prospective investigation of efficacy and safety.

## 1. Introduction

Chronic idiopathic constipation (CIC) is characterised by multiple symptoms and classified according to the Rome III criteria [1], with a half of patients typically suffering for 5 or more years [2]. CIC causes a significant reduction in health related quality of life (HRQOL) compared to the general population [3] in the community, HRQOL can be worse than inflammatory bowel disease in those attending secondary care [4], and the burden on healthcare resources is widely recognised [4–6]. Roughly 50% of patients are refractory to laxatives and lifestyle measures [7] and are often referred to secondary care where they have investigations including tests of transit and functional defecation disorder (FDD). A proportion of these cases are also refractory to drug therapy and biofeedback [8] and require further invasive therapy. Transanal irrigation (TAI, also commonly known as “rectal irrigation”) is a possible treatment for these patients.

TAI is a type of colonic irrigation which is self-administered by the patient at home after adequate training in the technique and which differs from commercialised colonic irrigation (hydrotherapy) only in the volume and length of time the water is left in situ. It typically involves transanal insertion of a rectal catheter or cone in order to instil lukewarm water retrograde into the colon. This is achieved through various commercially available irrigation systems which have either hand controlled or mechanical pumps, in a volume ranging from 500 mL to 1000 mL depending on patients' experience and tolerance. This is then drained naturally after a few minutes and can result in a satisfactory bowel movement.

The published literature provides very little evidence of efficacy in the CIC group and mostly reports effect in patients suffering faecal incontinence or constipation secondary to neurogenic bowel dysfunction (NBD) [9]. A systematic review of TAI studies reported successful treatment of constipation in 117/259 cases (45%) [10]. Of these 259 patients only 2 cohorts had a significant proportion with CIC in the mixed aetiology [11, 12] with numbers of 79 and 37 patients, respectively, and the remaining 143 patients mostly suffered neurogenic aetiology. The procedure has been extensively reported as simple to perform and relatively safe [11], with the estimated risk of the most serious complication (TAI induced colonic perforation) being less than 0.0002% per irrigation [9]. In 2013 an expert consensus review has specifically stated that there is an urgent need to evaluate effectiveness in the other conditions where TAI is an emerging treatment and if there are any physiological predictors of long-term response [9].

Patients who suffer from CIC are clearly a separate and distinctly different group of patients; the aetiology of the condition is completely unknown; there is evidence of its marked prevalence (14%) and chronicity [13] and subsequent detrimental effect on quality of life [4]. In this paper we aim to present retrospective evidence of the efficacy and safety of TAI in the largest reported cohort of tertiary care patients receiving this therapy for refractory CIC and use the prospective database these patients are enrolled on to identify any baseline predictors of efficacy.

## 2. Materials and Methods

Patients for this study were treated at the Durham Constipation Clinic (DCC), a tertiary referral centre in the North East of England receiving around 150 new patient referrals each year and with around 850 patients under follow-up. All patients prospectively give informed consent for enrolment onto an ethically approved database. This includes data of symptom and severity measures, physiological tests (transit, proctography, and physiology), and scores of symptom severity using the validated PAC-SYM [14] questionnaire.

The DCC team are experienced in using TAI as a minimally invasive technique for treating CIC after failed medical and behavioural therapies. Patients are selected for one of 3 differing forms of irrigation equipment based on patient choice and the ability of patients to manage the differing practical aspects of each type. Peristeen™ (Coloplast A/S®, Denmark), Qufora™ (MBH International A/S®, Denmark), and the Irrimatic pump™ (B. Braun Melsungen AG®, Germany) systems are all used, with the vast majority given the Peristeen™ system.

A service evaluation was designed to gain patient perspective on TAI as a treatment specific to refractory CIC. Patients under active clinic follow-up were identified through the prospective database and included if they fulfilled the Rome III criteria for functional constipation, had past or present treatment with TAI, and received TAI specifically for refractory CIC (failed all medical and behavioural therapies). Patients were excluded for any secondary causes of constipation (e.g., neurological or opioid use) or concomitant faecal incontinence. Evaluation of patients was not at specific time-points after commencement of therapy but was a snapshot of all patients who had used the therapy in our service within the preceding 12 months.

A 12-question form was designed for self-completion in clinic or via telephone interview by a team member. The form asked patients to indicate therapy commencement/cessation and total use, how they perceived response to the treatment, the number of irrigations performed on average each week, whether it improved particular CIC symptoms (stating yes or no if TAI improved), how satisfied they were with the therapy, and any adverse events or complications they encountered. Data was collected from February to June 2012. The duration of therapy was calculated from both participants answers on the actual length of time they had used it (to the nearest week) and notes entries on when it was started by the specialist nurse. The duration of therapy use was considered the main outcome measure as a surrogate marker of efficacy. The total number of irrigations for each person was calculated by participants reporting their frequency of use, which was then extrapolated over the therapy duration.

The prospective database of DCC patients' baseline assessments was used to identify baseline predictors of long-term TAI efficacy. Patients who had completed the service evaluation had their baseline data checked on the database and these were extracted and included in the analysis if present and consistent with investigation prior to commencing TAI. PAC-SYM score, transit study time, isotope proctogram, and barium proctogram results were included as relevant indicators of symptom severity and physiological profile of constipation. Transit time was calculated according to the day 4 time on the Metcalf protocol [15], and PAC-SYM mean item score as a validated outcome measure of symptom severity in CIC [14]. Proctograms were checked by team members and used to classify patients as having a functional defecation disorder (FDD) according to the Rome 3 criteria [1], by consensus opinion of 3 consultants. Where patients had both isotope and barium proctograms the measured evacuation percentage of the isotope proctogram was considered superior to the barium proctogram in classifying FDD. Data was analysed in SPSS in order to create a Kaplan-Meier survival curve of continuing use of TAI. The endpoint on the curve is defined as the duration of use until cessation of TAI due to lack of efficacy. Patients are censored on the curve where they continue with the therapy at their current duration of usage. Possible correlations between therapy duration and baseline measures were explored using scatter plots and Pearson's correlation. A Student's *t*-test was performed to assess for significant differences in length of therapy use between patients classified as FDD or non-FDD.

## 3. Results and Discussion

### 3.1. Results

**Figure 1:**
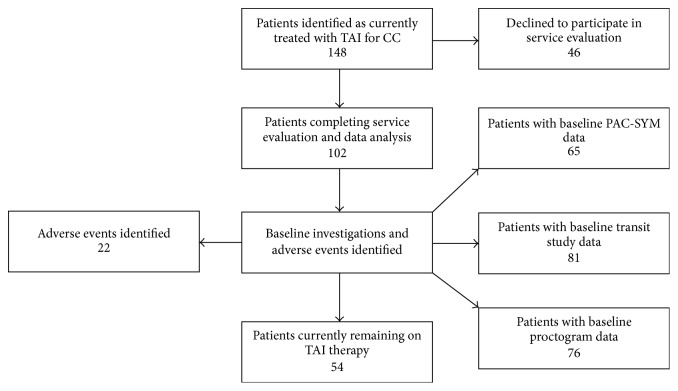
Patient flow.

148 people were identified (via database and specialist nurse records) and contacted, and 102 completed the service evaluation (69%) with consent for survey and demographic data analysis (Figure 1).  Table 1 demonstrates the demographics of the cohort. 53% of participants were currently still using TAI at data collection (Table 2), with a combined total of 21,476 irrigations reported over nearly 119 years of therapy use. Patients used TAI on average once every second day.

Participants reported “yes” or “no” if they believed that particular symptoms of CIC had been improved by TAI use (Table 3), with >42% reporting improvement in 4 of the 6 fields and >22% in all fields. Overall satisfaction with TAI (as a therapy) was reported by 67% of respondents as either moderately or very much better.

**Figure 2:**
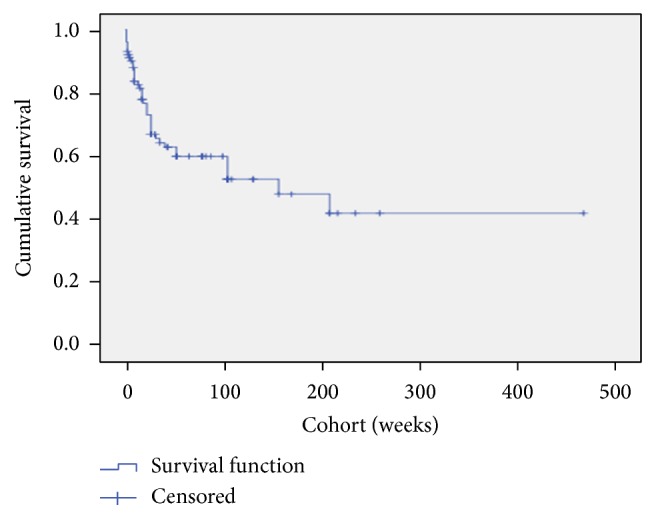
Kaplan-Meier survival of transanal irrigation. In this survival curve the endpoint was defined as patients' discontinuing TAI therapy due to perceived ineffectiveness. 40 patients reached the endpoint with 62 (60.0%) censored at their current duration of use of TAI. The curve demonstrates that a significant proportion of the cohort continue with the therapy.

**Figure 3:**
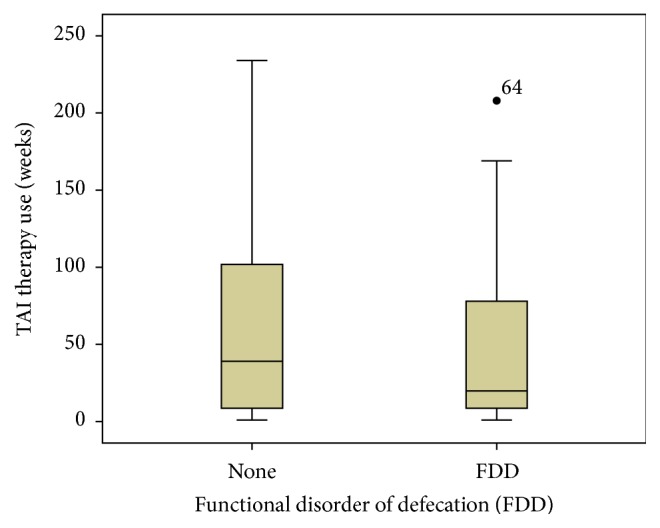
This boxplot compares the reported TAE therapy duration (*y*-axis, weeks) of patients classified from baseline proctograms as having a functional defecation disorder (FDD) [*N* = 40, mean use = 45 weeks, SD = 49.5] and no FDD [*N* = 36, mean use = 59 weeks, SD = 60.7], with no significant difference detected between these groups [*P* = 0.29].

In the Kaplan-Meier survival curve (Figure 2) it should be noted that 40 patients reached the endpoint with 62 (60.8%) censored at their current duration of use of TAI. The curve demonstrates that a significant proportion of the cohort continue with the therapy, although most are censored at their current duration of use within 2 years.  Table 4 outlines the baseline data collected from the prospective database on patient colonic transit times, PAC-SYM score, and categorisation of FDD through either isotope or barium proctogram (or both). A Pearson's correlation analysis did not demonstrate significant correlations in the duration of TAI use with any baseline demographics: age [*N* = 102, *r* = 0.018, *P* = 0.86], duration of CIC symptoms [*N* = 102, *r* = 0.033, *P* = 0.74], and frequency of TAI use [*N* = 102, *r* = −0.07, *P* = 0.48]. Similarly the baseline symptom severity and transit times did not demonstrate any correlation: mean item PAC-SYM [*N* = 65, *r* = −0.19, *P* = 0.13] and transit time [*N* = 81, *r* = −0.073, *P* = 0.52].  Figure 3 is a boxplot comparing the TAI therapy use of patients classified from baseline proctograms as having a FDD [*N* = 40, mean use = 45 weeks, SD = 49.5] and no FDD [*N* = 36, mean use = 59 weeks, SD = 60.7], with no significant difference in TAI therapy use (Student's *t*-test for equality of means) detected between these groups [*P* = 0.29].

Adverse events reported (Table 5) were relatively minor given the frequency and total duration of use, although 1 in 5 patients experienced one or more. Device and equipment problems were most frequently reported and minor medical complications such as rectal bleeding were easily managed by patients. A small proportion (2%) attributed new anal fissures to TAI usage. This series does not report any rectal or colonic perforations, and the unit has not experienced any of these major complications to date.

### 3.2. Discussion

Investigators across Europe have been reporting for 10 years that TAI is a beneficial treatment for patients suffering faecal incontinence and constipation due to a range of aetiologies [10–12, 16–18]. Our cohort is the largest reported who suffer specifically from CIC, with demonstrable chronicity (mean 21.8 years), symptom severity (mean item total PAC-SYM 2.23), and slow transit (mean 60.9 hrs), with a proportion suffering from FDD (53%).

The results in this cohort demonstrate that around 60% of patients with CIC use TAI for an extended period of time (1-2 years or more) and feel their symptoms are significantly improved. As this was a retrospective snapshot of outcomes, satisfaction rates at specific time intervals cannot be determined. However the duration of TAI therapy use is a justified outcome measure as this is a procedure which requires commitment and time (unlike drug treatments) and patients tend to discontinue ineffective treatments early. Duration of TAI therapy use is therefore a reasonable surrogate marker for efficacy.

The most severe symptoms of CIC (abdominal pain, bloating, incomplete emptying [of rectum], and bowel frequency) improved in over 42% of patients. Remarkably awareness of urge and spontaneous complete bowel movements occurred in a quarter and a fifth, respectively. No correlation was demonstrated between duration of TAI therapy use and patient age or duration of CIC, suggesting that the treatment can be just as effective in a patient who has suffered for 20 years as in someone with a six-month history. There was also no correlation between therapy duration and baseline transit time or presence of FDD suggesting that, on present evidence, the treatment can be offered to patients with any type of constipation. It might be a therapy that could be offered to community-assessed patients with only the treatment refractory referred for detailed investigations. A very slight negative correlation exists between baseline PAC-SYM severity and duration of therapy use: this does not reach statistical significance and merely reflects that patients with more severe symptoms fail treatments faster, which is hardly surprising.

There were no serious complications recorded despite over 20,000 irrigations used by this cohort. Indeed TAI has been used in our service for 7 years and the cohort studied probably represents less than a third of the total number of patients treated. We have not encountered any cases of rectal perforation or other serious complications in this time, though these problems have been reported and remain an unlikely possibility. A small proportion of patients developed new anal fissures during therapy but without a control group it is difficult to know if this is treatment related.

The exact mechanism through which TAI causes a bowel motion is unclear, although it is postulated to be due to stimulation or initiation of peristaltic waves through either stretching and/or warming the colon, and scintigraphic assessment has previously demonstrated washout to the splenic flexure [19]. This may explain the marked effects on chronic symptom improvements reported in our cohort. The spontaneous bowel movements between irrigations that occurred in a fifth of cases may suggest that the treatment has effects that go beyond a simple washout. One possible mechanism could be the initiation of a normal rectal urge to defecate, which is lost or diminished in around two-thirds of patients with chronic constipation [20] and may be due to raised sensorimotor thresholds [21]. An alternative hypothesis relates to the release of 5HT when colonic muscle is distended, thus increasing peristalsis [22].

Overall, a significant proportion of patients in this cohort are globally satisfied with the therapy, reporting marked symptom improvements and minimal complications. International expert consensus on the treatment algorithms of CIC in both Europe and the US has failed to adequately recognise the value and position of this treatment [23, 24].

## 4. Conclusions

Our results are retrospective, uncontrolled, and possibly affected by reporting bias as patients were interviewed by clinic staff and as such should be treated with caution. A prospective controlled multicentre study is therefore required together with assessments of cost-effectiveness and qualitative studies of the experience of the procedure.

These results do add weight to a body of evidence that TAI is an effective and safe minimally invasive treatment for CIC, either definitively in some or as a bridge to other treatments. For now, the medical ethos of “first do no harm” and common sense dictate that it should be considered as an option on the CIC treatment algorithm before any form of invasive abdominal surgery.

## Figures and Tables

**Table 1:** Baseline *demographics*.

Number of patients	102
Median age in years [range]	45 [25–84]
Number of females [%]	95 [93%]
Number of males [%]	7 [7%]
Mean duration of CIC in years [SD : SE]	21.8 [16.9 : 1.7]

SD = standard deviation, SE = standard error.

**Table 2:** Transanal irrigation *(TAI) therapy use*.

Presently still using TAI	54 [53%]
Completely stopped TAI	48 [47%]
Combined length of therapy weeks [years]	6,175 [118.8]
Mean length of therapy use in weeks [SD : SE]	60.5 [73.2 : 7.3]
Median length of therapy use in weeks [range]	30.15 [1–468]
Combined irrigations	21,476
Mean irrigations/week [SD : SE]	*3.7 [2.6 : 0.26]*

SD = standard deviation, SE = standard error.

**Table 3:** Symptom improvement and overall satisfaction with transanal irrigation (TAI).

*Symptoms improved*	
Bowel frequency	43 (42%)
Clearance of rectum	64 (63%)
Abdominal pain	49 (48%)
Bloating	50 (49%)
General well-being	66 (65%)
Awareness of urge	25 (25%)
Spontaneous complete bowel movements (SCBMs)	22 (22%)
*Overall satisfaction*	
No better	34 (33%)
Moderately better	*40 (39%)*
Very much better	28 (28%)

**Table 4:** Baseline investigations on prospective database (cohort).

Investigation	*N*	Result
Transit study mean time (hours) [SD : SE]	81	60.9 [15.6 : 1.7]
PAC-SYM mean total score [SD : SE]	65	2.23 [0.76 : 0.09]
Baseline isotope & barium proctograms classified to FDD or no	76	FDD 40
FDD by consensus of consultant coauthors		None 36

Standard deviation (SD), standard error (SE), functional defecation disorder (FDD).

**Table 5:** Adverse events (AEs).

All AEs	22/102 (21.6%)
*TAI devices*	
Bursting balloons	10 (9.8%)
Catheters splitting	3 (2.9%)
*Medical problems*	
Rectal bleeding	6 (5.9%)
Painful irrigations	3 (2.9%)
Painful haemorrhoids	2 (2.0%)
New anal fissure	2 (2%)
Perforation	0 (0%)

## Abbreviations

TAI: Transanal irrigation
CIC: Chronic idiopathic constipation
NBD: Neurogenic bowel dysfunction
FDD: Functional defecation disorder
DCC: Durham Constipation Clinic
HRQOL: Health related quality of life
PAC-SYM: Patient assessment of constipation symptoms.
